# Plastics are a new threat to Palau’s coral reefs

**DOI:** 10.1371/journal.pone.0270237

**Published:** 2022-07-06

**Authors:** Eric Béraud, Vanessa Bednarz, Ikelau Otto, Yimnang Golbuu, Christine Ferrier-Pagès

**Affiliations:** 1 Centre Scientifique de Monaco, Equipe Ecophysiologie Corallienne, Monaco, Monaco; 2 PICRC, Palau International Coral Reef Center, Koror, Palau, Republic of Palau; Newcastle University, UNITED KINGDOM

## Abstract

Plastic pollution of the oceans has long been an ongoing and growing problem. Single-use plastic (plastic bags and microbeads) is responsible for most of this pollution. In recent years, studies have highlighted the importance of the size of plastic particles, and the impact of this pollution source on the environment. We determined the concentration of small marine plastics in seawater, sediments and beach sand around a pristine reef area (Republic of Palau) using very simple tools (plankton net, sieves, organic matter degradation, density separation, Nile red fluorochrome). In this study, we succeeded in detecting microplastic (MPs) particles and microplastic fibers, but also nanoplastic (NPs). These three types of particles were found in all samples with a large heterogeneity, from 0.01 to 0.09 particles L-1 and 0.17 to 32.13 particles g-1 DW for MPs in seawater, sediments and sand, respectively. Even when NPs were identified, the amounts of NPs were underestimated and varied from 0.09 to 0.43 particles L^-1^ in seawater and from 1.08 to 71.02 particles g^-1^ DW in sediment and sand, respectively. These variations could be attributed to the environmental characteristics of the different sites. This study shows that plastic pollution must be considered in environmental studies even in the most pristine locations. It also shows that NPs pollution is related to the amount of MPs found at the sites. To understand the effects of this plastic pollution, it is necessary that the next toxicological studies take into account the effects of this fraction that makes up the NPs.

## Introduction

In recent decades, plastic pollution of the oceans has been steadily increasing and has become one of the major threats to marine life [1, 2]. Due to its chemical properties [3], plastic can persist for decades and continuously accumulate in the oceans. It is estimated that 4.8 to 12.7 million tons of plastic waste enter the ocean every year nowadays [4]. More than 92% of the plastic waste currently found in the ocean is microplastic (MPs), particles less than 5 mm in size [5]. MPs can pollute the marine environment either as primary or secondary MPs. Primary MPs enter the ocean directly in the form of microfibers from clothing, microbeads, and plastic pellets [6, 7], *while secondary MPs result from the degradation of larger plastic pieces* through mechanical abrasion and photochemical oxidation [7–9]. MPs can break down into even smaller pieces called nanoplastics (NPs: < 1 μm, [10, 11]. but the actual amount of nanoplastics in the ocean is still unknown. MPs have been shown to accumulate in all oceans and environments, from the sea surface to the deep sediments, and from the subtropical oceanic eddies to polar regions [12–14]. This is because plastic is initially buoyant, and is easily dispersed over long distances by wave action and wind [15]. Therefore, pristine environments are not protected from plastic pollution at all.

MPs can be ingested by almost all marine organisms, from invertebrates to whales due to their small size [16, 17]. The resulting effects range from severe injuries such as stomach abrasions and gut blockage [18], to reduced food intake and energy availability [19], a decline in fecundity and reproductive success [20, 21] and mortality of the organisms [22]. In addition to these physical damages, MPs often contain toxic plastic additives, adsorb persistent pollutants and heavy metals from seawater [1, 23], and are colonized by a variety of microorganisms including pathogens [24]. Thus, ingestion of MPs can transfer chemical pollutants and pathogens to marine organisms, exacerbating the adverse effects on marine organisms.

Although awareness of the harmfulness of plastic debris to marine life is increasing, knowledge of the abundance and size distribution of plastic debris in areas of low human impact is still very low [25–27]. This is true for coral reef ecosystems in pristine areas far from major urban locations and pollution sources. Coral reefs are one of the most biodiverse habitats on the planet, providing nursery, breeding, and feeding grounds for a wide variety of organisms [28]. Coral reefs are also vital in providing natural resources for humans [29]. The Palau Archipelago, consisting of about 340 islands in the northwestern Pacific Ocean, has pristine reefs that support some of the richest and most diverse marine life. However, Palau shares maritime boundaries with intensely populated countries such as Philippines, Indonesia and Micronesia, and is under the influence of the Indonesian Through Flow area, characterized by high commercial shipping activities, and possibly high plastic contamination zones. However, such contamination has not been investigated yet in the Palau area. Considering that the reefs of Palau, as most of the world’s reefs, are now facing several global (e.g. ocean warming and acidification) and local (e.g. eutrophication, terrestrial run-off) stressors, plastic contamination can be an additional threat for these fragile ecosystems. Thus, scientific research is needed to understand the extent to which MPs affect coral reef systems. The objective of this study was to investigate MP pollution in seawater, sediment, and beaches of the main islands of Palau, Babeldaob, Koror, and Rock Islands, using a protocol that can be easily implemented in reef areas.

## Materials and methods

### Ethics statement

This study was conducted in accordance with Palau International Coral Reef Center requirements for non-extractive research. This research did not involve any endangered or protected species and no animals were sampled.

### Study area and sampling sites

**Fig 1:**
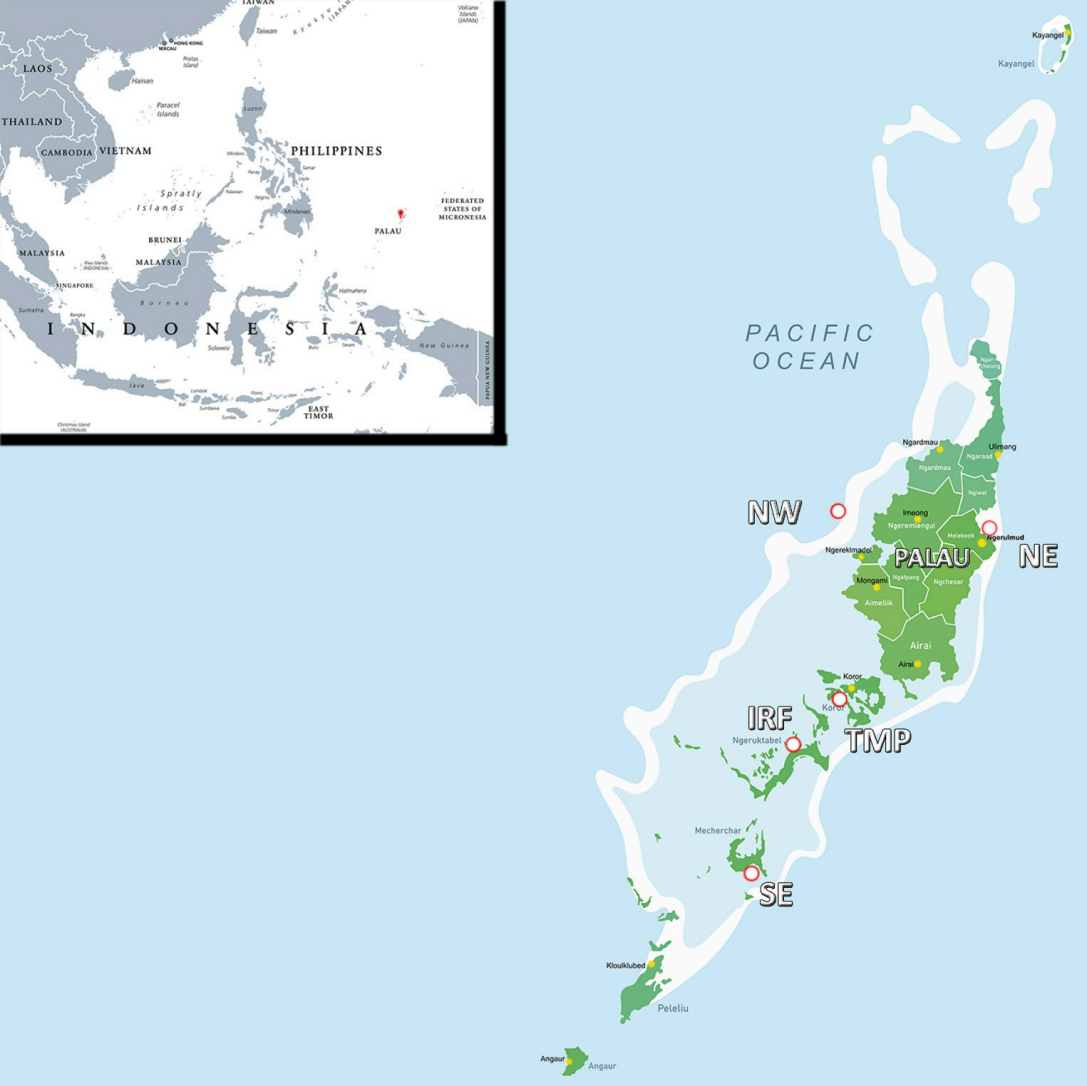
Map of the microplastic sampling sites around the main island Babeldaob and the Southern Rock Island Lagoon in the Palau archipelago.

Since 2001, the Palau International Coral Reef Center has established a long-term coral reef monitoring program at various sites throughout the archipelago. These sites include 26 Marine Protected Areas (MPAs) and Highly Protected Reserves (HPs), which currently cover a total area of 499 km2 and 65 km2, respectively. The five sampling sites selected for microplastic quantification in this study (Table 1, Fig 1) were all located in close proximity to the long-term monitoring stations to complement what was already being done as part of the Palauan monitoring effort. The sampling has been performed in collaboration with PICRC divers who have permanent collection permits. Three sites were located at the northwest (NW), northeast (NE), and southeast (SE) island-ocean interface of the main island of Babeldaob. There, reef sediment samples were collected from both the outer and inner reefs, while seawater samples were collected only from the outer reefs. These sites were located near a sand beach for sand sampling. The other two sites were located in the inner bays of the rocky islands to the south of the main town of Koror. There, only seawater and reef sediments were collected from the inner reefs, as there are no beaches or outer reefs. One of the inner reef sites (IRF) was located ~15 km from Koror and represents an uninhabited bay, while the other site (TMP) was located near Koror in close proximity to the outfall of the sewage treatment plant.

**Table 1:** Overview about the different sampling locations.

Sampling location	Surface seawater	Beach sediment	Reef sediment	Coordinates
1. North-West (NW)	Outer reef	yes	Outer + inner reef	Between N 07°31.413’ E 134°28.086’ and N 07°33.617’ E 134°29.993’
2. North-East (NE)	- ^a^	yes	Outer + inner reef	Between N 07°30.592’ E 134°37.961’ and N 07°31.186’ E134°38.151’
3. South-East (SE)	Outer reef	yes	Outer + inner reef	Between N 07°07.251’ E134°22.144’ and N 07°07.039’ E 134°21.627’
4. Inner bay (IRF)	Inner reef	- ^b^	Inner reef	N 07°17.464’ E 134°25.103’
5. Inner bay (TMP)	Inner reef	- ^b^	Inner reef	N 07°19.277’ E 134°26.932’

^a^ Seawater samples on the East side of Palau were only collected at the south-eastern site.

^b^ No beach sand was collected due to the absence of beaches.

### Sample collection

Surface samples of seawater were collected using a zooplankton trawl net with a relatively small size mesh (100 μm mesh size, 40 cm internal diameter, 1 m length,) compared to other large nets usually used in oceanographic cruises [30–32]. It was equipped with a flow meter (Hydro-Bios flow meter 438110). At each sampling station, the net was towed alongside the boat at a speed of 4 knots for 15 minutes (n = 6). A 2 kg weight was attached to the net and ensured that the net opening remained ~10 cm below the water surface during towing. The flow meter reading was recorded before and after each trawl to allow accurate calculation of the amount of water filtered. After each trawl, the net was returned to the boat and rinsed externally with a deck hose to concentrate the sample material in the cod end (100 μm mesh size). The cod end was removed and rinsed with filtered seawater to transfer the concentrated sample into 0.5-L glass bottles. All samples were stored in a cooler until further processing in the laboratory on the same day. In the laboratory, seawater samples were concentrated on a 10 μm sieve and the solid contents were deposited on a pre-weighed filter paper (diameter 15 cm, pore size 10–13μm, thickness 17 μm) using MilliQ water followed by vacuum filtration. All filter papers were then dried in an oven at 90°C for 24 hours. The weight of the filter with the dried sample was measured again to determine the dry weight of the sample. The filters were kept dry until being processed.

Subsurface sediment samples were collected from coral reefs by diving (SCUBA) rather than using a bottom grab. Although the technique described below by SCUBA is less accurate than using a grab, it requires less challenging material and can be easily performed by anyone. At each site, 0.3 m x 0.3 m quadrats (n = 6) were placed every 15 m along a 100 m transect line at approximately 13 m water depth. The top 5 cm sediment layer of each quadrat was carefully and slowly collected into glass tubes using a metal scoop. Care was taken not to agitate the sediment too much during sampling. After collection, each tube was sealed and taken to the laboratory for further processing. Note that sampling in the sediments on the outer reefs could only be done at 3 of the sites because the barrier reef is not present throughout Koror Island. In turn, beach sand samples (n = 6) were collected at each of the three beach sites every 15 m along a 100 m coastal transect by laying out 0.3 m x 0.3 m quadrats at the high tide line (i.e., the farthest extent of the most recent high tide level). Large pieces of natural debris were removed from each quadrat before the top layer of sediment (approximately 5 cm) was collected from each quadrat with a metal shovel. The collected material was stored in aluminum containers and transported to the laboratory the same day for further processing.

All reef sediment and beach sand samples were placed in aluminum containers and oven dried at 90°C for 2–3 days. The dried samples were ground into powder using a pestle and the dry weight was determined. Then, the samples were sieved on aluminum sieve of 5 mm to exclude particles > 5.0 mm from further analysis. The sediment fraction was weighed and then placed in a glass container for density separation. There, a saturated NaCl solution (density of 1,198 at 25°C) was added and the sample was continuously stirred for 10 minutes to facilitate the buoyancy of microplastic particles with a lower density than the NaCl solution. The solution was left overnight to allow the sedimentation of all particles except plastics [33]. The supernatant of each beaker was then collected on a 10 μm sieve, and the collected material was placed on a pre-weighed filter paper (diameter 15 cm, pore size 10–13μm, thickness 17 μm) using MilliQ water followed by vacuum filtration. All filter papers were subsequently dried in an oven at 90°C for 24 hours. The weight of the filter with the dried sample was measured again to determine the dry weight of the sample.

### Microplastic extraction from the filters

Care was taken not to use plastic items when processing samples. Personal protective equipment, lab coats, and gloves were worn at all times. Blank samples (without biological material or microplastics) were run in parallel with samples containing dried material, solutions, or trawled material. Blank samples were analyzed for MPs in the same manner as the other samples. The results obtained are < 1 NPs and < 0.2 MPs or fibers for airborne controls as for salt controls. In environmental samples, MPs may be embedded in organic matrices (covered with biofilms or attached to microorganisms) that need to be removed from the samples to optimize MP recovery. Here, the organic matter was degraded using the Fenton reaction:

$$Fe^{2+}{}_{\left(aq\right)}+H_{2}O_{2}\to Fe^{3+}{}_{\left(aq\right)}+OH^{-}{}_{\left(aq\right)}+HO^{•}$$

For this purpose, 0.15 g of each filter (from seawater, sediment and beach sand) was placed in a glass container with 2 mL of double distilled water and sonicated for 4 min. Then 5 mL each of a H_2_O_2_ (30%) and Fe II (0.05 M) solution were added. This step was repeated until the organics were completely degraded. To complete the reaction, the samples were placed in a dry bath at 55°C overnight. The next day, a concentrated NaCl solution was added to the solution and heated to 70°C in a dry bath to flush out the microplastic particles. After stirring for 30 minutes, the solution was centrifuged at 2500 g for 30 minutes. The supernatant was subsequently filtered onto black 0.2 μm isopore filter and treated with 5 mL of a Nile red solution (0.01 mg L-1) for 10 minutes. The filter was then placed in an oven at 45°C in the dark for 20 minutes before the orange-stained microplastic particles were counted under a microscope.

### Microplastic quantification

Microplastics were counted using an epifluorescence microscope (Leica DMI-4000, UV light, I3 filter: excitation/emission 450–490 / 515 long pass; Leica Microsystem) using a counting grid of 100 small squares with known surface area. Under the excitation light, the microplastics covered with Nile red appear orange. Depending on abundance and size, the plastics were counted either on 60 squares (100x magnification) or on several whole grids (100 squares). A proportional conversion factor accounting for the surface area of the squares relative to the total surface area of the filter was then applied to calculate the total number of plastics found on the filter. MPs (size > 1 μm) were divided into two categories: fibers and other microplastics, grouped together as MP-fragments. In addition, orange colored particles on the filter with size < 1 μm were also counted under the microscope and labeled as NPs. Indeed, some NPs were found on the filters, which could have come from the initial aggregates of organic matter retained on the filter. The amount of MPs and NPs was normalized to the dry weight of the sample (for sand and sediment DW) or the volume of water (for seawater).

### Statistical analysis

Statistical analysis was performed using R v3.2.2 (R Development Core Team, 2015). All data were checked before to meet assumption for parametric tests using Shapiro-Wilk test. Univariate one-way analysis of variance (ANOVA) was used, followed by Tukey’s HSD post hoc test, to compare the plastic concentrations within and between sites and differences were significant for p values < 0.05.

## Results

### Microplastic abundance in the studied areas

**Fig 2:**
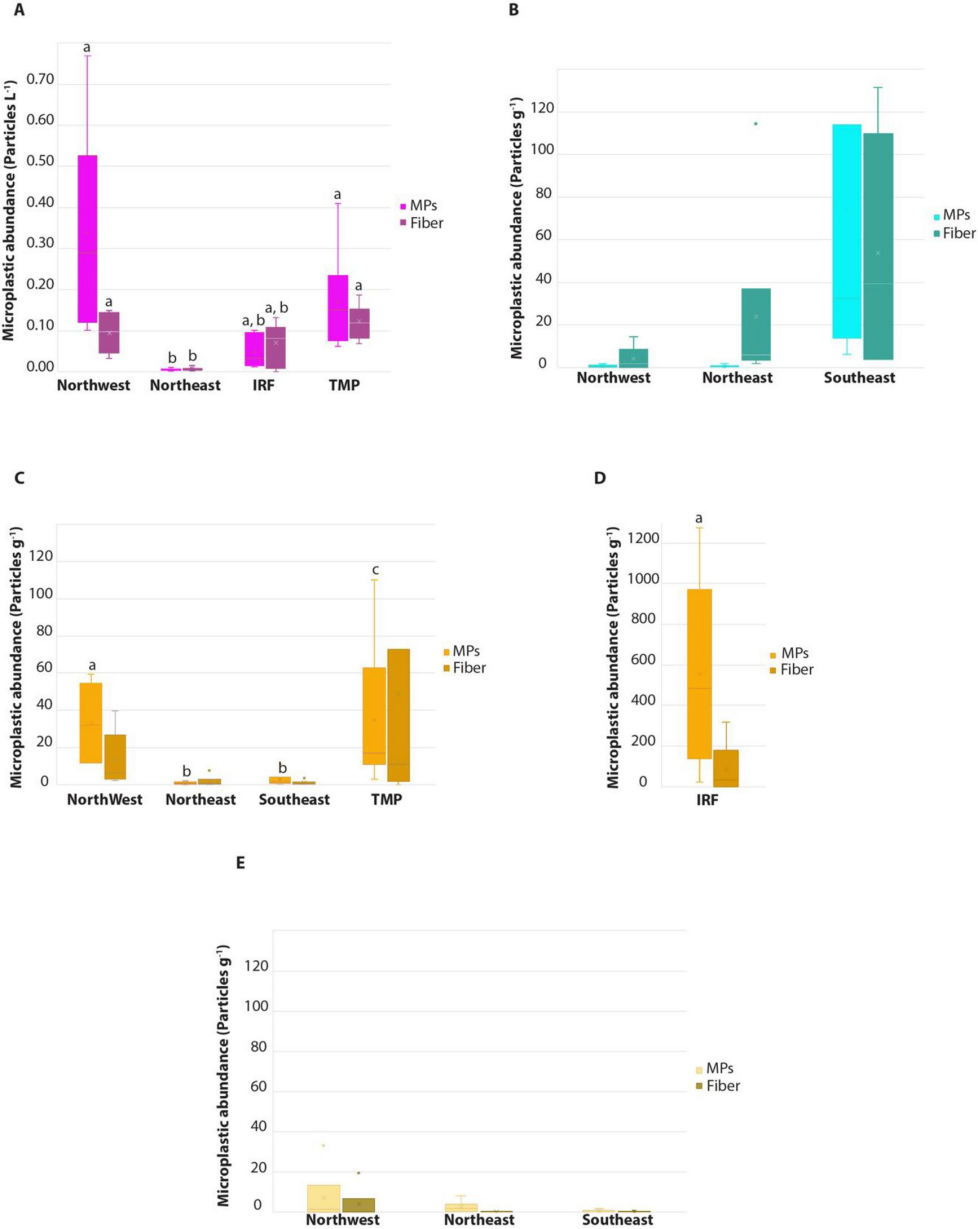
Microplastic abundance from the 5 different sites investigated, Northwest, Northeast, Inner Reef (IRF), wastewater treatment plant (TMP). A: surface seawater, B: sediments from the outer barrier reef, C & D: sediments from the inner barrier reef, E: Beaches. Light colors: plastic particles between 100 microns and 5 mm, Dark colors: plastic fibers. Median values are represented with a line in the boxplots, the average with a cross and outliers with dots. Different letters above the columns indicate significant differences between sites for MP-fragments and fibers separately (one way ANOVA followed by Tukey’s HSD test): p < 0.05.

The abundance of MP-fragments in seawater (Fig 2A) ranged from 0.01 to 0.09 particles L^-1^ (average 0.05 ± 0.04 particles L^-1^). The lowest amount of MP-fragments was detected in the water at the Northeast coast (0.01± 0.00 items L^-1^), while Northwest and TMP sites had significantly higher MP-fragment abundances (p = 0.0059). MP-fragments were more abundant than fibers in the Northwest and TMP sites (78.1 and 58.90% of total MPs, respectively, p < 0.05). Conversely, fibers were slightly more abundant than MP-fragments on the Northeast and IRF sites (respectively 54.43 and 59.59% of total MPs).

Concerning the subsurface sediment samples, both the inner reef and outer reef sediments were considered. For the sediments of the outer barrier reefs (Fig 2B), the Southeast location had similar quantities of both MP-fragments and fibers (ca. 6 total particles g^-1^ DW) and the MP-fragment concentration was significantly higher (p = 0.0314) compared to the other two locations, (< 2 total particles g^-1^ DW). In the Northwest and Northeast sites, fibers were more dominant than MP-fragments and accounted for 95 and 75% of the total MPs, respectively.

For the inner reefs, sediments from the IRF site, presenting 12.71 ± 8.92 MP-fragments g^-1^ DW, were significantly more enriched in MP particles than the sediments from the other sites (Fig 2C and 2D; p < 0.094). Also, sediments from the Northeast site contained the lowest amount of MPs fragments than all other sites (0.11 ± 0.0.08 MPs g^-1^ DW, p < 0.001). No significant difference in MP concentrations was detected among the other sites. In addition, concentrations of fibers (g^-1^ DW sediment) were not different between sites (Fig 2C and 2D, p > 0.05). Overall, we observed differences in the relative percentage of MP fragments and fibers among sites. MP-fragments tended to be more abundant than fibers at all sites, except for the northeast site, where fibers contributed for 55% of the plastic contamination.

The beach sands (Fig 2E) contained significantly less MP-fragments and fibers compared to the corresponding reef sediments, with a total of only 0.17 to 0.34 particles g^-1^ DW (p < 0.004). The lowest amount of particles was detected at the east coast (with respectively 0.22± 0.10 and 0.17± 0.09 particles g^-1^ DW for the south and the north sites), while the Northwest site had significantly higher particle abundance (0.34 ± 0.13 particles g^-1^ DW; p <0.03). Overall, MP-fragments were significantly more abundant than fibers among all studied sites accounting between 77 to 89% of all detected microplastics (p < 0.038).

### Unexpected results: Nano-plastic particles

Overall, NPs were present at all studied sites (Table 2). They were heterogeneously distributed among the studied sites, ranging from 0.1 to 0.43 items L^-1^ seawater and from 1.08 to 71 particles g^-1^ DW (for sediments and beaches). The two eastern sites (Northeast and Southeast) tended to have lower quantities of NPs than the western site (Northwest), with the exception made for the sediments, which presented the highest concentration of NPs with 8.76 ± 5.62 particles g^-1^ DW (p <0.05). In the north (both sites) the trend is towards a concentration of NPs from offshore to onshore (outer reef sediments < inner reef sediments < beaches). This trend was more pronounced for the west coast as compared to the east coast (sediment of the inner barrier reef and beaches had respectively 5.7 and 3 times more NPs in the west than the east coasts). At the Southeast site, however, there was an inverse trend, since NPs were more abundant in the outer barrier reef sediments than in the inner barrier reef sediments, and beaches.

**Table 2:** Nanoplastic abundance in seawater (particles L^-1^), sediments and beaches (particles g^-1^ DW) from 5 different sites. Northwest, Northeast, Inner Reef (IRF), wastewater TreatMent Plant (TMP), Southeast. Different letters indicate significant differences among sites (one way ANOVA followed by Tukey’s HSD test): p < 0.05.

	Northwest		Northeast		IRF		TMP		Southeast	
	Mean	SD	Mean	SD	Mean	SD	Mean	SD	Mean	SD
**Seawater**	0.25^a,b^	0.12	0.09^a^	0.04	0.18^a,b^	0.24	0.43^b^	0.18		
**Outer reef sediments**	3.39^a,b^	2.36	1.50^b^	1.54					8.76^a^	5.62
**Inner reef sediments**	6.12^a^	4.04	1.08^b^	0.71	71.02^c^	29.31	6.76^a^	2.24	2.02^a,b^	2.66
**Beaches**	8.45^a^	4.58	2.84^a^	2.12					3.93^a^	3.08

Although only a very small percentage of their total abundance has been caught with our sampling procedures, NPs were much more abundant than MPs (fragments and fibers combined) at all 4 studied sites (n = 6, p = 0.02). They represented between 54.9 and 96.1% of the total plastic particles found in the investigated areas.

NPs concentrations were correlated with fibers (from 74.6 to 99.9%) and MP-fragment concentrations (99.9 to 95.9%), as observed in the outer barrier reef sediment from the south (8.76 ± 5.62 particles g^-1^ DW), inner barrier reef sediment from the IRF (71.02 ± 29.31 particles g^-1^ DW) as well as in the sand from the northwest beaches (8.45 ± 4.58 Particles g^-1^ DW). The largest quantities of MP-fragments and NPs are found on the IRF site with 12.71 ± 8.92 particles g^-1^ DW and 71.02 ± 29.31 particles g^-1^ DW, respectively.

## Discussion

This study highlights significant pollution from MPs in a pristine reef area, far away from the hotspots of urbanization and vessel traffic. This finding is consistent with the fact that plastic particles are ubiquitous in the marine environment and have been found even in the most pristine areas of the planet, including the deep sea and polar regions [34]. MP contamination in Palau suggests that it depends more on seawater currents than on the proximity of anthropogenic activities, as is often the case in other areas [35–38]. Most importantly, our study pinpoints significant, but still under-estimated NPs pollution.

MPs distribution presented a great heterogeneity between all sites investigated, in agreement with the observations from other environments [22, 39]. This is probably due to the fact that the MPs are mainly derived from fragmentation of larger plastics, whose distribution is heterogeneous due to their large size. Nevertheless, the comparison of seawater and sediment pollution in Palau with other pristine and non-pristine areas (S1 and S2 Tables in S1 File) suggests that Palau’s reefs are not protected from plastic pollution. However, the comparisons are affected by the different mesh sizes of the plankton nets and the different protocols used to estimate plastic concentrations at the different sites. For example, concentrations of MPs in surface waters of the remote, uninhabited coral reefs of the Nansha Islands on the western border of the Coral Triangle were 0.0556 ± 0.0355 items m^-3^ [40]. These concentrations are lower than those found in Palau’s waters (a mean of 0.05 particles L^-1^ or 50 particles m^-3^) and could be due to the larger mesh size used in Nansha (333 μm) compared to the present study (100 μm). The same can be observed with the study of Bakir et al. (2020) [41], in which MP concentrations are lower, but the mesh size used is larger. Concentrations in surface waters of North America were also higher than those measured in Palau (0.53 items L^-1^), but again, with a larger mesh size (333 μm), which may have underestimated the actual pollution there [42]. On the contrary to the above studies, the highest concentrations recorded in the Netherlands coastal area (10 to 187 particles L^-1^) were obtained with a very small mesh size of 10 μm [19]. Concerning the beaches of Palau, the MP concentrations (ca. 1.89 to 3.78 particles m^-2^) obtained with a 10 μm sieve appear very low compared to those of Henderson Island, a remote, uninhabited island in the South Pacific, (671.6 items m^-2^). Although a very large mesh size (2–5 mm) was used for this study, this seems to indicate that remote islands near oceanic plastic accumulation zones act as important sinks [43]. Finally, the sediments in Palau are, with the exception of IRF site (5 times more concentrated), in the same range of MPs-fragments concentrations obtained in Europe or in North America, which present only 0.2 and 2.7 particles g^-1^ DW (sieved on >32 μm or > 250 μm; [42]). Overall, these comparisons suggest that there is an urgent need to develop a standardized methodology that allows for long-term monitoring of MPs in the oceans. For example, most studies (including this one) have used plankton nets with mesh sizes much larger than the size of MPs (S1 and S2 Tables in S1 File), which naturally leads to underestimation of plastic pollution. This problem is highlighted by the lower plastic pollution in the Nansha Islands, where a net of 333 μm was used, compared to the Palau Islands, where a net of 100 μm was used. So far, it is very difficult to find large plankton nets with a small mesh size, and vendors have to take this problem into account. The mesh size of the sieves used for sediment and sand beach should also take into account the small size of the MPs. Although it is difficult to directly sieve sand on a sieve of 1 μm, sieves of different sizes can be used subsequently. Protocols must take into account that many reef areas, for example, are located in remote areas with limited access to large vessels or modern technology. The protocol proposed in this study has the advantage of being easy to implement in remote areas but can be improved in view of recent protocols. For example, drying samples at high temperature (90°C) may degrade plastics if a step of elutriation is used [44], and a slightly lower temperature (60°C), with a longer drying period might be more adapted. Besley et al. (2017) [45] also found that multiple extractions in NaCL were required to recover all plastics, also found that multiple extractions in NaCL were required to recover all plastics, while [46] found that the majority of microplastics were extracted after the first extraction. The number of extractions may depend on the quantity and quality of sediment used to extract plastics, as well as the settling time used to separate the plastics from the sediment. In light of the above studies, we recommend using a settling time > 6h as in this study and performing tests for the number of extractions required.

Plastic fibers are a special type of microplastics since they come essentially from clothing manufacturing and productive tools (fishing nets, ropes, and lines, etc.) [35, 47, 48]. The fiber concentrations in the surface seawater around Palau (3 to 40 particles m^-3^), are in the lower end of the concentration range found in the Western pacific (0.15 to 450 particles m^-3^) [49, 50]. Fibers were also detected in similar amounts in all sediment samples. The presence of fibers in sediments from the TMP site (49 particles g^-1^ DW) was expected due to the proximity of this site to the waste treatment plant outfall and because cloth washing is considered one of the major contributors of fibers to the environment [37]. The fiber contamination of sediments from the Western outer reefs (up to 53 particles g^-1^ DW) and Eastern outer reefs (24 to 54 particles g^-1^ DW) can rather be due to the fishing activity within this 85896 sq km area.

The first report of NPs occurring in ocean surface samples has been provided only 3 years ago [51]. In this study, NPs were detected by GC-MS pyrolysis of environmental matrices. Our simple protocol for detecting microplastic contaminants also retained the fraction of NPs that was certainly embedded in large fragments of organic matter. Because all samples were first sieved through a 10–100 μm mesh before collection, particles smaller than 100 μm were not retained. Therefore, the total concentration of NPs measured in this study is greatly underestimated, and yet, already represents significant contamination in all areas sampled. Such high contamination is not surprising since the number of plastic particles increases exponentially with decreasing size [52]. Therefore, Ter Halle et al. (2017) [51] have estimated, based on mass conservation principles, that NPs fragmentation would lead to concentrations that are ultimately 10^14^ times higher than the currently found MPs concentrations. As expected, the presence of NPs was strongly correlated to the presence of MPs (92.3 to 83.8%), and fibers (from 88.5 to 98.1%). This observation suggests that the NPs are either subject to the same environmental constrains than the MPs (as described above), or are derived from microplastic fragmentation, except maybe at the TMP site, where the NPs may have been released directly by the wastewater treatment plant [37].

**Fig 3:**
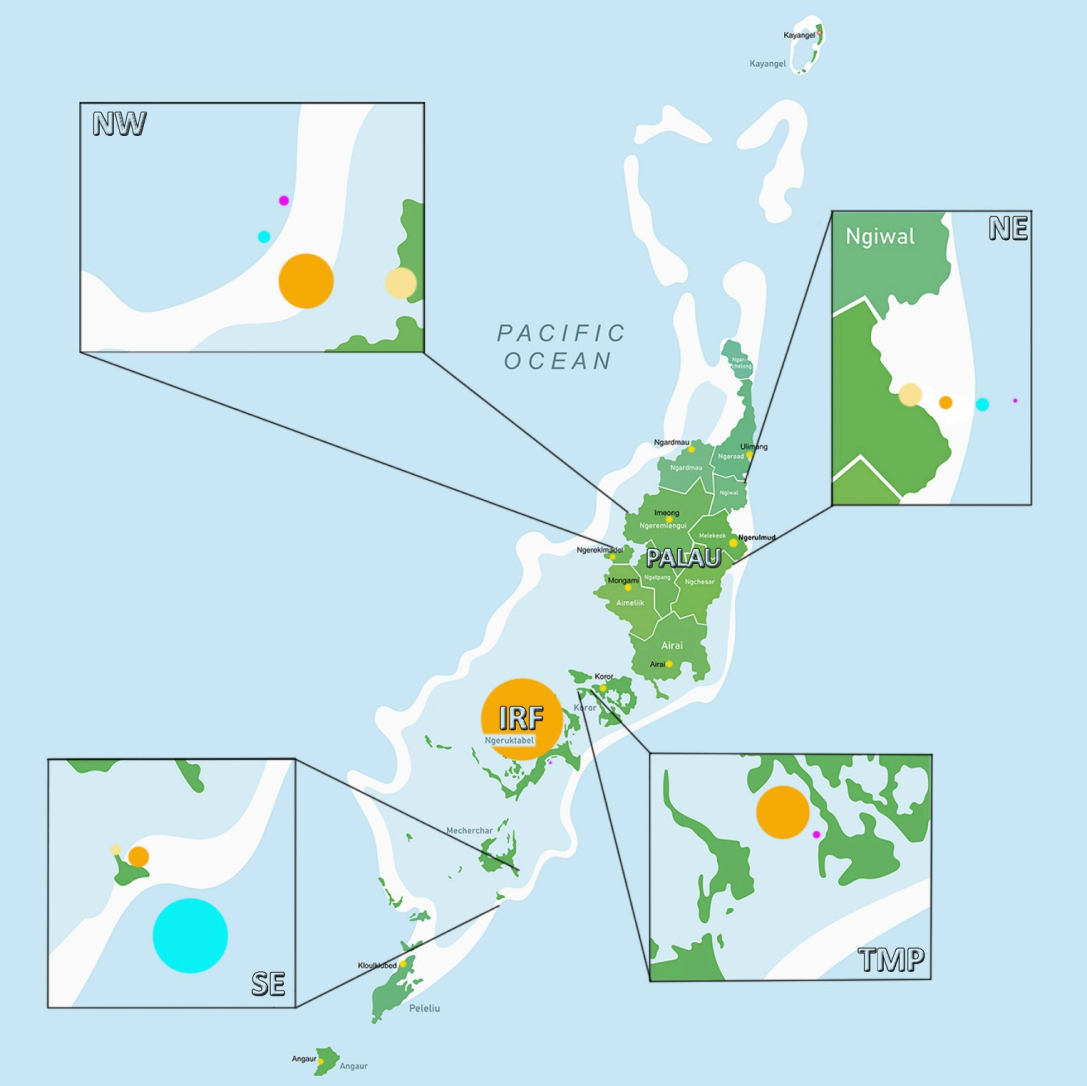
Map of the representation of the quantity of MPs found according to the type of sample. Blue: Outer sediments from the barrier reef, Purple: water sample, Orange: Inner sediments from the barrier reef, Yellow: Beach sample.

**Fig 4:**
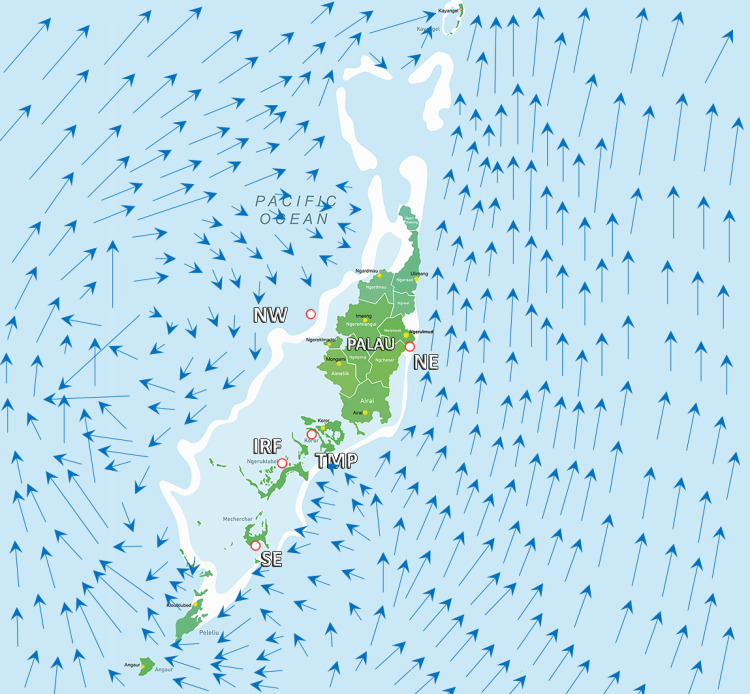
Model of currents around the island from the southeast, showing the effect of island mass on currents (modified from [76]).

Taking into account the microplastic concentrations in the different locations of Palau (Fig 2), we produced a map (Fig 3), which highlights a spatial distribution in plastic contamination, with a greater microplastic pollution in the Southeast, IRF and TMP as compared to the other investigated areas of the island. Differences in ocean current patterns, reef structures, and/or the proximity of a city can explain the observed MPs distribution. The Southeast is a remote location with respect to urbanization. The observed gradient of plastic pollution here, which decreases from the outer reefs to the inner reefs and beaches, suggests that pollution comes from the water and penetrates into the lagoon. This hypothesis is in agreement with the measured current patterns (Fig 4), which clearly show the presence of a gyre in this zone, with a strong flow entering into the lagoon. Such current pattern can also explain the large plastic contamination within the sediment of the IRF, where plastics can be trapped and sink to the bottom in this calmer area following a slow process of continuous sedimentation of the small quantities of plastic contained in surface waters. Sedimentation of MPs can be enhanced during rainy periods, which decreases salinity as well as the buoyancy of plastic particles [53]. The MPs contamination of the TMP site is more likely due to the wastewater treatment plant, which is known to be a major source of MPs at other locations [53–56]. The northeast contained the lowest amount of MPs, possibly due to the fact that ocean currents are not directed toward the coast, but transport plastic particles further north and offshore (Fig 4). The MP pollution gradient is also reversed compared to the southeast, with beaches being more contaminated than internal sediments and seawater. This gradient can be explained by the presence of a barrier reef along the coast, whose large coral plateau acts as a pollution sink, especially at low tide. There, the plastics are highly exposed to UV-induced degradation and are then washed onto the beaches as the tide rises. The role of the coral plateau as a plastic sink is confirmed at the northwest site, which has a larger reef plateau as well as higher plastic concentrations in the sediment of the inner barrier reef. Compared to the northeast, this site is also exposed to stronger currents towards the coast and has well-developed mangroves that can act as a second plastic sink by trapping plastics in their rich organic material (colloid) content. Overall, there are several factors that may explain the observed differences in MP concentrations in Palau. These include the direction of seawater currents that may carry MP to shore, the presence of reef plateaus and mangroves that act as barriers and sinks, and the presence of a sewage treatment plant as a source of MP pollution.

The extent to which reef organisms are being exposed to MPs and NPs depends on the actual contamination. Sediments highly contaminated with plastics (MPs and NPs) might directly affect the sediment-dwelling organisms and the corals [57], before being spread to the whole pelagic food web, via predation of the organisms and/or sediment re-suspension [58]. Since sediments are located after the reef flat, our results show that the reef acts as a plastic trap, which will be thus an additional threat to corals [24, 59–62] and other reef organisms [63]. Corals and other filter feeders will also be exposed to plastics contained in seawater [20]. Although it is difficult to compare plastic contamination in seawater and sediment due to different normalization units, and although plastics in seawater are restricted to the first 5m depth [64], seawater gets filtered a lot by organisms, which may thus take up MPs from seawater easier than MPs from the sediment. Nevertheless, both seawater and sediment will act in combination, to impact the health of reef organisms. Both MPs and NPs have various effects on aquatic organisms, including incorporation into living tissues, oxidative stress, toxicity and enhancement of immune responses [6, 65, 66]. In addition, toxic additives may leach from plastics into the environment [67]. Plastics can also adsorb and accumulate organic and inorganic pollutants from seawater that may contaminate organisms when plastics are ingested [68–72]. The expected pollution with NPs in the marine environment is expected to largely increase over time as they are continuously applied in a variety of consumer products [73], represent by-products of several manufacturing processes and derive from the degradation of macro- and microplastics [53, 74, 75]. Plastic pollution in the reef waters and sediments of Palau, which is not a hotspot of human activity, needs to be considered in future studies, especially during heat waves, which in combination with plastic pollution can particularly affect coral reef organisms.

## Supporting information

S1 File (DOCX)
