# Supplementary material for: Plastics are a new threat to Palau’s coral reefs
Source: PLoS One. 2022 Jul 6;17(7):e0270237. doi: 10.1371/journal.pone.0270237 (PMC9258895; doi:10.1371/journal.pone.0270237)
Supplement: S1 File — (DOCX) [file pone.0270237.s001.docx]

**Plastics are a new threat to Palau’s coral reefs**

Béraud Eric^1*^, Bednarz Vanessa^1^, Otto Ikelau^2^, Golbuu Yimnang^2^, Christine Ferrier-Pagès^1^

Supplementary information

| **Location** | **Technique used** | | | | | **MPs**  **(m^-3^)** | **Source** |
| --- | --- | --- | --- | --- | --- | --- | --- |
| ***Pacific Ocean*** |  | | |  | |  |  |
| ***North Pacific*** | | | | |  |  |  |
| Bering Sea | Ring net | Diam : 1,3m ; 0.3mm | | | | 0.000016 | Day & Shay (1987) |
| Subartic N.P. |  |  |  |  |  | 0.00067 |  |
| ***Eastern North Pacific*** | |  | | | |  |  |
|  |  |  | | | |  |  |
| Vancouver Island, Canada | Pumping (- 4.5m) | 500-62.5 μm | | | | 279 | Desforges *et al.* (2014) |
| N.P. Central Gyre | Manta net | 333 μm | | | | 2.23 | Moore *et al.* (2001) |
|  |  |  |  |  |  | 0.017 | Carson *et al.* (2013) |
| Santa Monica Bay,  California, USA | Manta net | 333 μm | | | | 3.92 | Lattin *et al.* (2004) |
|  |  |  |  |  |  | 7.25 | Moore *et al.* (2002) |
| N.P. subtropical gyre | Manta net | 333 μm | | | | 0.0042-0.089 | Goldstein *et al.* (2013) |
| ***Western North Pacific*** | |  | | | |  |  |
|  |  |  | | | |  |  |
| Subtropical N.P. | Ring net | 0.3mm | | | | 0.019 | Day & Shay (1987) |
| Kuroshio current System | Neuston Net | 330 μm | | | | 0.034 | Yamashita & Tanimura (2007) |
| Yangtze estuary system, East China Sea | Neuston Net | 333 μm | | | | 4137.3 | Zhao *et al.* (2014) |
| Geoje Island,  South Korea | Hand net, Manta net | 50 μm 333 μm | | | | 16000 | Song *et al.* (2014) |
| Nansha Island  South china sea | Manta net | 333 μm | | | | 0.0556 | Tan *et al.* (2020) |
| ***South Pacific*** | |  | | | |  |  |
|  |  |  | | | |  |  |
| South Pacific  subtropical gyre | Manta net | 333 μm | | | | 0.0054 | Eriksen *et al.* (2013) |
| Vanuatu | Manta net | 333 μm | | | | 0.05-0.57 | Bakir et al (2020) |
| Fidji  Laucala Bay  Vanua Navakavu  Suva Harbour | Plancton net | 125 μm | | | | 0.09  0.24  0.1 | Ferreira *et al.* (2020) |
| Australian coast | Manta net | 333 μm | | | | 0.00085 | Reisser *et al.* (2013) |
| ***Atlantic Ocean*** |  |  | | | |  |  |
| ***North Atlantic*** | |  | | | |  |  |
| North Atlantic gyre | Plancton net | 335 μm | | | | 0.0041 | Law *et al.* (2010) |
| ***Northwest Atlantic*** | |  | | | |  |  |
|  |  |  | | | |  |  |
| Gulf of Maine | Plancton net | | 335 μm | | | 0.00031 | Law *et al.* (2010) |
| ***Caribbean Sea*** | | |  | | |  |  |
|  |  |  |  | | |  |  |
| Caribean | Plancton net | | 335 μm | | | 0.00028 | Law *et al.* (2010) |
| ***Northeast Atlantic*** | |  | | | |  |  |
| Offshore, Ireland | Underway  sampling | 250 μm | | | | 2.46 | Lusher *et al.* (2014) |
| English Channel,  U.K. | Plankton net | 200 μm | | | | 0.27 | Cole *et al.* (2014a) |
| ***Equatorial Atlantic*** | |  | | | |  |  |
|  |  |  | | | |  |  |
| St. Peter and St. Paul  Archipelago, Brazil | Plankton net | 300 μm | | | | 0.01 | Ivar do Sul *et al.* (2013) |
| ***South Atlantic*** | |  | | | |  |  |
|  |  |  | | | |  |  |
| Cape Province,  South Africa | Neuston Net | 0,9 mm | | | | 0.00073 | Ryan (1988) |
| Fernando de Noronha,  Abrolhos and Trindade, Brazil | Manta net | 300 μm | | | | 0.03 | Ivar do Sul *et al.* (2014) |
| Gioana estuary, Brazil | Conical plancton net | 300 μm | | | | 0.26 | Lima *et al.* (2014) |
| ***Mediterranean and European seas*** | |  | | | |  |  |
|  |  |  | | | |  |  |
| West coast, Sweden | Manta net | (80 μm) | | | | 150–2400 | Norén (2007) |
|  |  | (450 μm) | | | | 0.01–0.14 |  |
| Northwest  Mediterranean | Manta net | 333 μm | | | | 0.27 | Collignon *et al.* (2012) |
| Bay of Calvi,  Corsica, France | wp2 net | 200 μm | | | | 0.15 | de Lucia *et al.* (2014) |
| Gulf of Oristano,  Sardinia, Italy | Manta net | 500 μm | | | | 0–0.74 | Magnusson (2014) |

**Table 1:** Mean microplastic abundance in surface waters. When concentrations were reported per m^2^ or km^2^, data were converted per m^-3^ taking into account, for a proxy of depth, the first 20 cm of water/sediment, where the plastic particles accumulate. After Lusher 2015.

| **Location** | **Plastic types** | | | | **MPs** | | | **Source** | |
| --- | --- | --- | --- | --- | --- | --- | --- | --- | --- |
| ***North Pacific*** | | |  | |  |  |  |  |  |
|  |  |  |  | |  | | |  | |
| Kauai, Hawaiian islands | Fragments and pellets 0.8–6.5 mm | | | | / | | | Corcoran *et al.* (2009) | |
|  | Fragments <1 cm | | | | / | | | Cooper and Corcoran  (2010) | |
| ***Northeast Pacific*** | |  | | |  | | |  | |
|  |  |  | | |  | | |  | |
| San Diego, California,  USA | Pellets and fragments <5 mm | | | | / | | | Van *et al.* (2012) | |
| ***Northwest Pacific*** | |  | | |  | | |  | |
|  |  |  | | |  | | |  | |
| Coastal beaches, Russia | Fragments and pellets | | | | 29 m^-2^ | | | Kusui and Noda (2003) | |
| Coastal beaches, Japan | Pellets <5 mm | | | | >100 per beach | | | Endo *et al.* (2005) | |
| Coastal beaches, Chile | Fragments and pellets 1-10 mm | | | | 30 m**^-^**^2^ | | | Hidalgo-Riz and Thiel (2013) | |
| Easter Island, Chile |  |  |  |  | 805 m**^-^**^2^ | | |  |  |
| ***SouthPacific*** | |  | | |  | | |  | |
|  |  |  | | |  | | |  | |
| Henderson Island | Fragments 2-5 mm | | | | 671.6m^-2^ | | | Lavers & Bond (2017) | |
| Vanuatu, Port Vila  Salomon Island, Honiara | >2 μm | | | | | | 333-33300 kg**^-^**^1^  450-15167 kg**^-^**^1^ | | Bakir et al (2020) |
| Fidji,Laucala Bay,  Vanua Navakavu,  Suva Harbour | >300 μm | | | | | | 19.8 kg**^-^**^1^ | | Ferreira *et al.* (2020) |
| ***Atlantic Ocean*** |  |  | | |  | | |  | |
| ***North Atlantic*** | |  | | |  | | |  | |
| Nova Scotia, Canada | Fibers 63-500 μm | | | | 200 - 800 fibres kg**^-^**^1^ | | | Mathalon and Hill (2014) | |
| *Maine, USA | Pellets and fragments 250 μm | | | | 105 kg**^-^**^1^ | | | Graham and Thompson (2009) | |
| *Florida, USA |  |  |  |  | 214 kg**^-^**^1^ | | |  |  |
| Florida Keys, USA | Pellets and fragments 2-5 mm | | | | 100 - 1000 m^-2^ | | | Wilber (1987) | |
| Cape Cod, USA |  |  |  |  |  |  |  |  |  |
| North Carolina, USA | Fragments <5 mm | | | | 60 % of 14747 debris | | | Viehman *et al.* (2011) | |
| Bermuda | Pellets and fragments 2-5 mm | | | | 2000 - 10000 m^-2^ | | | Wilber (1987) | |
| Bahamas |  |  |  |  | 200 – 1000 m^-2^ | | |  |  |
| Lesser Antilles |  |  |  |  | 50 – 5000 m^-2^ | | |  |  |
| Portuguese coast | Pellets and fragments <5 mm | | | | 185.1 m^-2^ | | | Martins and Sobral (2011) | |
| Canary Islands, Spain | Pellets and fragments <5 mm | | | | >40 g kg^-1^ | | | Baztan *et al.* (2014) | |
| ***English Channel*** | |  | | |  | | |  | |
|  |  |  | | |  | | |  | |
| Tamar estuary, U.K. | Fragments <1 mm | | | | 65 % of 952 debris | | | Browne *et al.* (2010) | |
| ***South Atlantic*** | | |  | |  | | |  | |
|  |  |  |  | |  | | |  | |
| Recife, Brazil | Pellets 3.3 % of 2661 debris | | | | 300000 m^-3^ | | | Costa *et al.* (2010) | |
| ***Mediterranean and European*** | | | |  | |  | | |  |
| ***North Sea*** | |  | | |  | | |  | |
| Industrial harbor sediment, Sweden | Pellets > 80 μm | | | | 3220 kg^-1^ | | | Norén (2007) | |
| Industrial coastal sediment, Sweden |  |  |  |  | 340 kg^-1^ | | |  |  |
| Spiekeroog, Germany | Fibers and granules> 1.2 μm | | | | 3800 kg^-1^ d.w. | | | Liebezeit and Dubaish (2012) | |
| Jade System, Germany | Fibers and granules > 40 μm | | | | 64 - 88 kg^-1^ | | | Dubaish and Liebezeit (2013) | |
| *Harbor, Belgium | Fibers and granules > 38 μm | | | | 116.7 kg^-1^ d.w. | | | Claessens *et al.* (2011) | |
| *Continental shelf, Belgium |  |  |  |  | 97.2 kg^-1^ | | |  |  |
| Beach, Belgium |  |  |  |  | 92.8 kg^-1^ | | |  |  |
| Beach, Belgium | Pellets and fragments > 35 μm | | | | 17 kg^-1^ | | | Van Cauwenberghe *et al.* (2013a) | |
| ***Mediterranean sea*** | |  | | |  | | |  | |
|  |  |  | | |  | | |  | |
| Venice lagoon, Italy | Fibers and granules > 32 μm | | | | 672 - 2175 kg^-1^ d.w. | | | Vianello *et al.* (2013) | |
| Kea Island, Greece | Pellets 4-1mm | | | | 10 – 575 m^-2^ | | | Kaberi *et al.* (2013) | |

**Table 2:** Mean microplastic abundance in sediments. All sediments are beach sediments unless annotated with *, which refers to benthic or subtidal sediment. After lusher 2015.

**References**

Bakir A, Desender M, Wilkinson T, Van Hoytema N, Amos R, AIrahui S, et al. Occurrence and abundance of meso and microplastics in sediments, surface waters and marine biota from the South Pacific region. Mar Pollut Bull. 2020;160: 111572. doi: 10.1016/j.marpolbul.2020.111572.

Baztan J, Carrasco A, Chouinard O, Cleaud M, Gabaldon JE, Huck T, et al. Protected areas in the Atlantic facing the hazards of micro-plastic pollution: First diagnosis of three islands in the Canary Current. Mar Pollut Bull. 2014;80: 302–311. doi: 10.1016/j.marpolbul.2013.12.052.

Browne MA, Galloway TS, Thompson RC. Spatial patterns of plastic debris along estuarine shorelines. Environ Sci & Tech. 2010;44: 3404–3409. doi: 10.1021/es903784e.

Carson HS, Nerheim MS, Carroll KA, Eriksen M. The plastic-associated microorganisms of the North Pacific Gyre. Mar Poll Bull. 2013;75: 126–132. doi: 10.1016/j.marpolbul.2013.07.054.

Claessens M, Van Cauwenberghe L, Vandegehuchte MB, Janssen, CR. New techniques for the detection of microplastics in sediments and field collected organisms. Mar Poll Bull. 2013;70: 227–233. doi: 10.1016/j.marpolbul.2013.03.009.

Cole M, Webb H, Lindeque PK, Fileman ES, Halsband C, Galloway TS. Isolation of microplastics in biota-rich seawater samples and marine organisms. Sci Rep. 2014a;4: 4528. doi: 10.1038/srep04528.

Collignon A, Hecq JH, Glagani F, Voisin P, Collard F, Goffart A. Neustonic microplastic and zooplankton in the North Western Mediterranean Sea. Mar Pollut Bull. 2012;64: 861–864. doi: 10.1016/j.marpolbul.2012.01.011.

Cooper DA, Corcoran PL. Effects of mechanical and chemical processes on the degradation of plastic beach debris on the island of Kauai, Hawaii. Mar Pollut Bull. 2010;60: 650–654. doi: 10.1016/j.marpolbul.2009.12.026.

Corcoran PL, Biesinger MC, Grifi M. Plastics and beaches: A degrading relationship. Mar Pollut Bull. 2009;58: 80–84. doi: 10.1016/j.marpolbul.2008.08.022.

Costa MF, Ivar JA, Christina M, Ângela BA, Paula S, Ivar do Sul JA, et al. On the importance of size of plastic fragments and pellets on the strandline: a snapshot of a Brazilian beach. Environ Monit Assess. 2010;168: 299–304. doi: 10.1007/s10661-009-1113-4.

Day RH, Shaw DG. Patterns in the abundance of pelagic plastic and tar in the North Pacific Ocean, 1976–1985. Mar. Pollut. Bull. 1987;18: 311–316. doi: 10.1016/S0025-326X(87)80017-6.

De Lucia G, Caliani I, Marra S, Camedda A, Coppa S, Alcaro L, et al. Amount and distribution of neustonic micro-plastic off the Western Sardinian coast (Central-Western Mediterranean Sea). Mar Environ Res. 2014;100: 10–16. doi: 10.1016/j.marenvres.2014.03.017.

Desforges JPW, Galbraith M, Dangerfield N, Ross PS. Widespread distribution of microplastics in subsurface seawater in the NE Pacific Ocean. Mar Pollut Bull. 2014;79: 94–99. doi: 10.1016/j.marpolbul.2013.12.035.

Dubaish F, Liebezeit G. Suspended microplastics and black carbon particles in the Jade System, Southern North Sea. Water Air Soil pollut. 2013;224: 1–8. doi: 10.1007/s11270-012-1352-9.

Endo S, Takizawa R. Okuda K, Takada H, Chiba K, Kanehiro H, et al. Concentration of polychlorinated biphenyls (PCBs) in beached resin pellets: Variability among individual particles and regional differences. Mar Pollut Bull. 2005;50: 1103–1114. doi: 10.1016/j.marpolbul.2005.04.030.

Eriksen M, Maximenko N, Thiel M. Plastic pollution in the South Pacific subtropical gyre. Mar Pollut Bull. 2013;68: 71–76. doi: 10.1016/j.marpolbul.2012.12.021.

Hidalgo-Ruz V, Thiel M. Distribution and abundance of small plastic debris on beaches in the SE Pacific (Chile): A study supported by a citizen science project. Mar Environ Res. 2013;87: 12–18. doi: 10.1016/j.marenvres.2013.02.015.

Ferreira M, Thompson J, Paris A, Rohindra D, Rico C. Presence of microplastics in water, sediments and fish species in an urban coastal environment of Fiji, a Pacific small island developing state. Mar Pollut Bull. 2020;153: 110991. doi: 10.1016/j.marpolbul.2020.110991.

Goldstein MC, Titmus AJ, Ford M. Scales of spatial heterogeneity of plastic marine debris in the Northeast Pacific Ocean. PLoS ONE. 2013;8: 11 e80020. doi:10.1371/ journal.pone.0080020.

Graham ER, Thompson JT. Deposit- and suspension-feeding sea cucumbers (Echinodermata) ingest plastic fragments. J Exp Mar Biol Ecol. 2009;368: 22–29. doi: [10.1016/j.jembe.2008.09.007](https://doi.org/10.1016/j.jembe.2008.09.007).

Ivar do Sul JA, Costa MF, Barletta M, Cysneiros FJA. Pelagic microplastics around an archipelago of the Equatorial Atlantic. Mar Pollut Bull. 2013;75: 305–309. doi: 10.1016/j.marpolbul.2013.07.040.

Ivar do Sul JA, Costa MF, Fillmann G. Microplastics in the pelagic environment around oceanic islands of the Western Tropical Atlantic Ocean. Water Air Soil Pollut. 2014;225: 1–13. doi: 10.1007/s11270-014-2004-z.

Kaberi H, Tsangaris C, Zeri C, Mousdis G, Papadopoulos A, & Streftaris N. Microplastics along the shoreline of a Greek island (Kea island, Aegean Sea): Types and densities in relation to beach orientation, characteristics and proximity to sources. In Proceedings of the 4th International Conference on Environmental Management, Engineering, Planning and Economics (CEMEPE) and SECOTOX Conference; 2013. Mykonos island, Greece, June 24–28, ISBN:978-960-6865-68-8.

Kusui T, Noda M. International survey on the distribution of stranded and buried litter on beaches along the Sea of Japan. Mar Pollut Bull. 2003;47: 175–179. doi: 10.1016/S0025-326X(02)00478-2.

Lattin GL, Moore CJ, Zellers AF, Moore SL, Weisberg SB. A comparison of neustonic plastic and zooplankton at different depths near the southern California shore. Mar Pollut Bull. 2004;49: 291–294. doi: 10.1016/j.marpolbul.2004.01.020.

Lavers JL, Bond AL. Exceptional and rapid accumulation of anthropogenic debris on one of the world’s most remote and pristine islands. PNAS. 2017;114: 6052-6055. doi: 10.1073/pnas.1619818114.

Law KL, Morét-Ferguson S, Maximenko NA, Proskurowski G, Peacock EE, Hafner J, et al. Plastic accumulation in the North Atlantic subtropical gyre. Science. 2010;329: 1185–1188. doi: 10.1126/science.1192321.

Liebezeit G, Dubaish F. Microplastics in beaches of the East Frisian Islands Spiekeroog and Kachelotplate. Bull. Environ. Contam. Toxicol. 2012;89: 213–217. doi: 10.1007/s00128-012-0642-7.

Lima ARA, Costa MF, Barletta M. Distribution patterns of microplastics within the plankton of a tropical estuary. Environ Res. 2014;132: 146–155. doi: 10.1016/j.envres.2014.03.031.

Lusher A. Microplastics in the Marine Environment: Distribution, Interactions and Effects. In: Bergmann M., Gutow L., Klages M. (eds) Marine Anthropogenic Litter. Springer, Cham. 2015. doi: 10.1007/978-3-319-16510-3_10.

Lusher AL, Burke A, O’Connor I, Officer R. Microplastic pollution in the Northeast Atlantic Ocean: validated and opportunistic sampling. Mar Pollut Bull. 2014;88: 325–333. doi: 10.1016/j.marpolbul.2014.08.023.

Magnusson K. Microlitter and other microscopic anthropogenic particles in the sea area off Rauma and Turku, Finland. Swedish Environmental Institute Report 2014; U4645, 17 pp. Available from: <http://www.rauma.fi/ymparisto/ymparisto/U4645%20Microlitter%20RaumaTurku.pdf.>

Martins J, Sobral P. Plastic marine debris on the Portuguese coastline: A matter of size? Mar Pollut Bull. 2011;62: 2649–2653. doi: 0.1016/j.marpolbul.2011.09.028.

Mathalon A, Hill P. Microplastic fibers in the intertidal ecosystem surrounding Halifax Harbor, Nova Scotia. Mar Pollut Bull. 2014;81: 69–79. doi: 10.1016/j.marpolbul.2014.02.018.

Moore CJ, Moore SL, Leecaster MK, Weisberg SB. A comparison of plastic and plankton in the north Pacific central gyre. Mar Pollut Bull. 2001;42: 1297–1300. doi: 10.1016/s0025-326x(01)00114-x.

Moore CJ, Moore SL, Weisberg SB, Lattin GL, Zellers AF. A comparison of neustonic plastic and zooplankton abundance in southern California’s coastal waters. Mar Pollut Bull. 2002;44: 1035–1038. doi : 10.1016/s0025-326x(02)00150-9.

Norén F. Small Plastic Particles in Coastal Swedish Waters. N-Research report, commissioned by KIMO, Sweden. 2007; 11 pp.

Reisser J, Shaw J, Wilcox C, Hardesty BD, Proietti M, Thums M, et al. Marine plastic pollution in waters around Australia: Characteristics, concentrations, and pathways. PLoS ONE, 2013;8: 11 e80466. doi: 10.1371/journal.pone.0080466.

Ryan PG. The characteristics and distribution of plastic particles at the sea-surface off the southwestern Cape Province, South Africa. Mar Environ Res. 1988;25: 249–273. doi: 10.1016/0141-1136(88)90015-3.

Song Y K, Hong S H, Kang J H, Kwon O Y, Jang M, Han G M, et al. Large accumulation of micro-sized synthetic polymer particles in the sea surface microlayer. Environ Sci & Tech. 2014;48: 9014–9021. doi: 10.1021/es501757s.

Van A, Rochman CM, Flores EM, Hill KL, Varges E, Vargas SA, et al. Persistent organic pollutants in plastic marine debris found on beaches in San Diego, California. Chem. 2012;86: 258–263. doi: 10.1016/j.chemosphere.2011.09.039.

Van Cauwenberghe L, Claessens M, Vandegehuchte MB, Mees J, Janssen CR. Assessment of marine debris on the Belgian continental shelf. Mar Pollut Bull. 2013a;73: 161–169. doi: 10.1016/j.marpolbul.2013.05.026.

Vianello A, Boldrin A, Guerriero P, Moschino V, Rella R, Sturaro A, et al. Microplastic particles in sediments of Lagoon of Venice, Italy: First observations on occurrence, spatial patterns and identification. Estuar Coast Shelf Sci. 2013;130: 54–61. doi: 10.1016/j.ecss.2013.03.022.

Viehman S, Vander JL, Schellinger J, North C. Characterization of marine debris in North Carolina salt marshes. Mar Pollut Bull. 2011;62: 2771–2779. doi: 10.1016/j.marpolbul.2011.09.010.

Wilber RJ. Plastic in the North Atlantic. Oceanus, 1987;30: 61–68. Available from: <http://www.globalgarbage.org/plastic_in_the_north_atlantic_r._jude_wilber_oceanus_1987.pdf>

Yamashita R, Tanimura A. Floating plastic in the Kuroshio Current area, western North Pacific Ocean. Mar Pollut Bull. 2007;54: 485–488. doi: 10.1016/j.marpolbul.2006.11.012.
